# The role of national carbon pricing in phasing out China's coal power

**DOI:** 10.1016/j.isci.2021.102655

**Published:** 2021-05-27

**Authors:** Jianlei Mo, Weirong Zhang, Qiang Tu, Jiahai Yuan, Hongbo Duan, Ying Fan, Jiaofeng Pan, Jian Zhang, Zhixu Meng

**Affiliations:** 1 Center for Energy and Environmental Policy research, Institutes of Science and Development, Chinese Academy of Sciences, Beijing, 100190, China; 2 School of Economics and Management, North China Electric Power University, Beijing, 102206, China; 3 School of Finance, Tianjin University of Finance and Economics, Tianjin, 300222, China; 4 Beijing Key Laboratory of New Energy and Low-Carbon Development (North China Electric Power University), Changping, Beijing, 102206, China; 5 School of Economics and Management, University of Chinese Academy of Sciences, Beijing, 100190, China; 6 School of Economics and Management, Beihang University, Beijing, 100191, China; 7 School of Public Policy and Management, University of Chinese Academy of Sciences, Beijing, 100049, China

**Keywords:** energy system, energy economics, climate policy

## Abstract

As the country with the world's largest coal power capacity, China is launching a national carbon market. How the carbon pricing may contribute to phasing out China's coal power is a great concern. We collect full-sample data set of China's 4540 operating coal plant units and develop a stochastic Monte-Carlo financial model to assess the financial sustainability of the plant operation. Although China's coal plants have long residual technical lifetime, their operations are close to the break-even state. Even with low carbon price of 50 CNY/tCO_2_ growing at 4%/y and the permits being fully auctioned, the average residual lifetime of all the plants will be reduced by 5.43 years, and the cumulative CO_2_ emission from 2020 to 2050 will be reduced by 22.73 billion ton. The spatial disparity in the carbon pricing effect is significant, and the western regions are more vulnerable to the carbon pricing risk than the eastern regions.

## Highlights

• A bottom-up evaluation is conducted by making a unit-by-unit full-sample analysis
• Carbon pricing can substantially impact the financial sustainability of coal power
• Carbon permit allocation method plays a key role in determining the overall effect
• The spatial disparity in the carbon pricing effect is significant

## Graphical abstract

**Figure:**
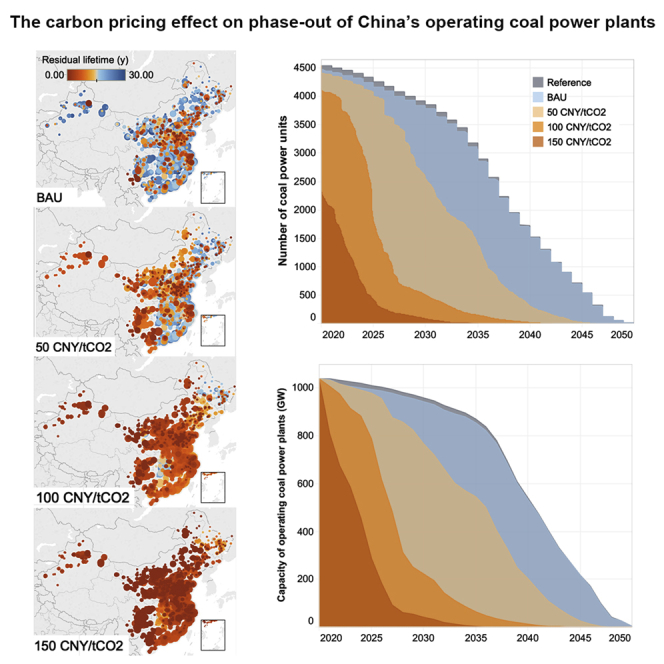

## Introduction

To keep the global mean temperature rise well below 2°C above the preindustrial levels and pursue efforts to limit it to 1.5°C, there needs to be a substantial decline in unabated coal power in the next decade and complete cessation by around 2050 (Luderer et al., 2018; https://www.ipcc.ch/site/assets/uploads/sites/2/2019/05/SR15_SPM_version_report_LR.pdf.). Especially for developing countries relying heavily on coal power, it is urgent to take additional policy measures to restrict coal power expansion and accelerate the phase-out of operating coal power plants. Although an agreement on the market mechanism at the global level still remains to be reached, carbon pricing, e.g., carbon tax or carbon market, is being increasingly implemented by regional, national and subnational jurisdictions, and 61 carbon pricing initiatives are already in place or are scheduled for implementation (World Bank, 2020). In this situation, carbon-intensive coal power plants will inevitably be exposed to carbon pricing risk (Sen and Von Schickfus, 2020), and some may even become decommissioned before their normal end of lifetime (Generation Foundation, 2013; IEA, 2013; World Bank, 2014; van der Ploeg and Rezai, 2020). Quantifying the carbon pricing effect on the phase out of coal power and carbon emission can provide practical implications for reshaping future energy investment strategies (Millar et al., 2018) and improving policy design to accelerate energy transition and achieve net zero carbon emission in power sector.

China, as the country with the world's largest coal power capacity, is attempting to build its nationwide carbon market, which may provide an impetus to phase out coal power and achieve its carbon peaking and carbon neutral targets early (Jakob et al., 2020). Specifically, China currently has more than 1,000 GW of coal power, which accounts for about 50% of global coal power capacity, and 62% of its electricity is produced by coal plants, which contributes more than 40% of its energy-related CO_2_ emissions (IEA, 2018), and, to a large extent, will determine the trend of China's future carbon emission evolution. The implementation of the national carbon pricing means that coal plant managers may have to pay for carbon emissions from electricity production, leading to higher generation costs, and they may suffer losses in future and even be forced to decommission coal plants before the normal end of plants' technical lifetime.

China's first carbon emission trading pilot was kicked off in Shenzhen from 2013, following which the other pilots in Beijing, Shanghai, Guangdong, Tianjin, Hubei, and Chongqing were launched. Based on the experiences from the pilots above, the national carbon market was announced in December 2017 (NDRC, 2017), and then it took three years to finish the capacity building, including the reporting, registration and trading system construction, and make trial run of the system. From 2021 the nationwide carbon pricing will be first implemented in China's power sector and gradually expanded to other sectors (Zhang et al., 2019; MEE (Ministry of Ecological Environment), 2020). For the coal power sector, the most relevant issues are the carbon emission permit allocation method and the carbon price level, which directly determine the additional operation costs and affect the cash flow and profits of the coal plant operation. In the practice of carbon emission trading, the carbon emission permits can be allocated to the entity by free allocation, partial auction, or full auction (Goulder et al., 2010). Although more than 90% of the emission permits are allocated freely in the pilot stage, it's expected that the ratio of the auctioned permits in the nationwide carbon market will increase gradually, in view of the international experience from the European Union emission trading scheme (EU ETS).

**Figure 1:**
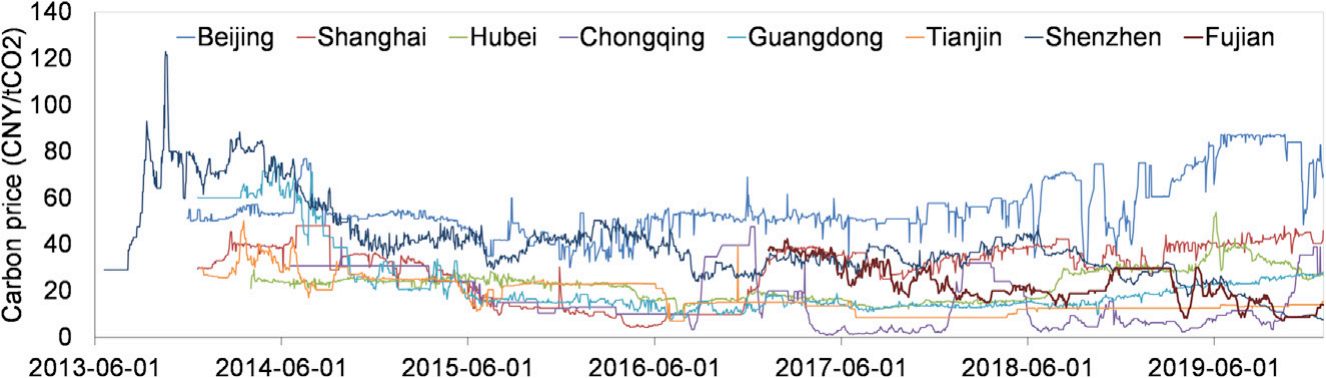
The carbon price evolution in China's local carbon markets

As the emission trading pilots are located in different regions, whose economy developments are at different stages, the difference in the carbon mitigation costs may be significant. Moreover, these pilots are not allowed to trade carbon emission permits with each other in the initial stage, thus it can be expected that the carbon prices in different pilots may be different from each other. The historical carbon prices from 2013 to 2019 in the local carbon markets are shown in Figure 1, and they vary from near zero to about 130 CNY/tCO_2_, reflecting the differences in the carbon mitigation costs as well as the carbon mitigation targets in these regions. Although the current price levels are lower than that in the initial stage overall, ranging from 10 CNY/tCO_2_ to 90 CNY/tCO_2_, the carbon price volatility are reduced and the price evolution become more stable. With the unified nationwide carbon pricing being implemented, the carbon prices in these regions are expected to converge to some common level, which may probably fall between 0 and 150 CNY/tCO_2_ in the near future based on the experiences of the pilots. In addition, the future carbon prices are expected to grow with the carbon mitigation target becoming more stringent in future, which reflects the facts that the carbon mitigation may become more difficult with carbon mitigation opportunities being exploited and the carbon mitigation cost may become higher (Morris et al., 2019). In this work, we aim to explore how the national carbon pricing would contribute to phasing out China's coal power and reducing carbon emission.

There are some relevant studies focusing on the required premature retirement of coal power to achieve climate targets of 1.5 or 2°C (Johnson et al., 2015; Pfeiffer et al., 2018; Cui et al., 2019; Wang et al., 2020), or conversely, the effect of the premature retirement of coal power on CO_2_ emission reductions and the achievement of climate targets (Jewell et al., 2019; Smith et al., 2019; Tong et al., 2019). However, the policy options and policy design for scheduling and enforcing coal plant phase-out are lacking and still need to be explored (Jakob et al., 2020). In addition, these studies have generally relied on integrated assessment models to conduct top-down analyses of the link between the long-term climate targets and the required phase-out of coal power at the global or national level, and implicitly assume that the coal power is phased out following the rules of cost effectiveness or a uniform reduction in the lifetime of all coal plants. However, the heterogeneity among the coal power plants at the micro-level and, especially, plant managers' operation decision regarding whether and when to decommission a plant based on the financial performance have been overlooked (Mo et al., 2021).

To fill these gaps, we develop a model framework using real-option-based Monte-Carlo methods to assess the financial sustainability of the operating coal plants (Dixit and Pindyck, 1994), based on which whether and when to decommission the operating coal plants under carbon pricing is explored. To take the heterogeneity among coal plants into full account, we construct a full-sample database consisting of detailed technical and economic data associated with the investment and operation of 4,540 operating coal power plant units, accounting for 1038 GW, i.e., nearly 100% of China's total operating coal power capacity. Then, a bottom-up evaluation of the carbon pricing effect on the coal plant operation is conducted by a unit-by-unit analysis, and by aggregating the results of the 4,540 plant units together, when and how much of the operating coal plant stock will be phased out by carbon pricing is obtained. Furthermore, in view of the heterogeneity among different regions in China, the disparity of the carbon pricing effect on the coal plant phase-out among China's 29 provinces, autonomous regions, and municipalities is explored, and the regions that are more vulnerable to the carbon pricing risk are identified.

## Results

To explore the carbon pricing effect on the phase-out of coal power, three policy scenarios are designed, i.e. Reference scenario, business as usual (BAU) scenario, and carbon pricing scenario. In the Reference scenario, the residual lifetime of the coal plants will not be affected by the economic and policy conditions, and all the plants will continue operating until the end of their technical lifetime; in the BAU scenario, the decision to decommission coal plants and accordingly the coal plants' residual lifetime will be affected by the existing market and policy conditions, but the carbon pricing policy is not considered; in the carbon pricing scenario, the plant operation will be affected by the fuel market, electricity market and especially the carbon pricing, and the plant lifetime would be shortened accordingly.

### The impact of carbon pricing on the operating coal plants' residual lifetime

**Figure 2:**
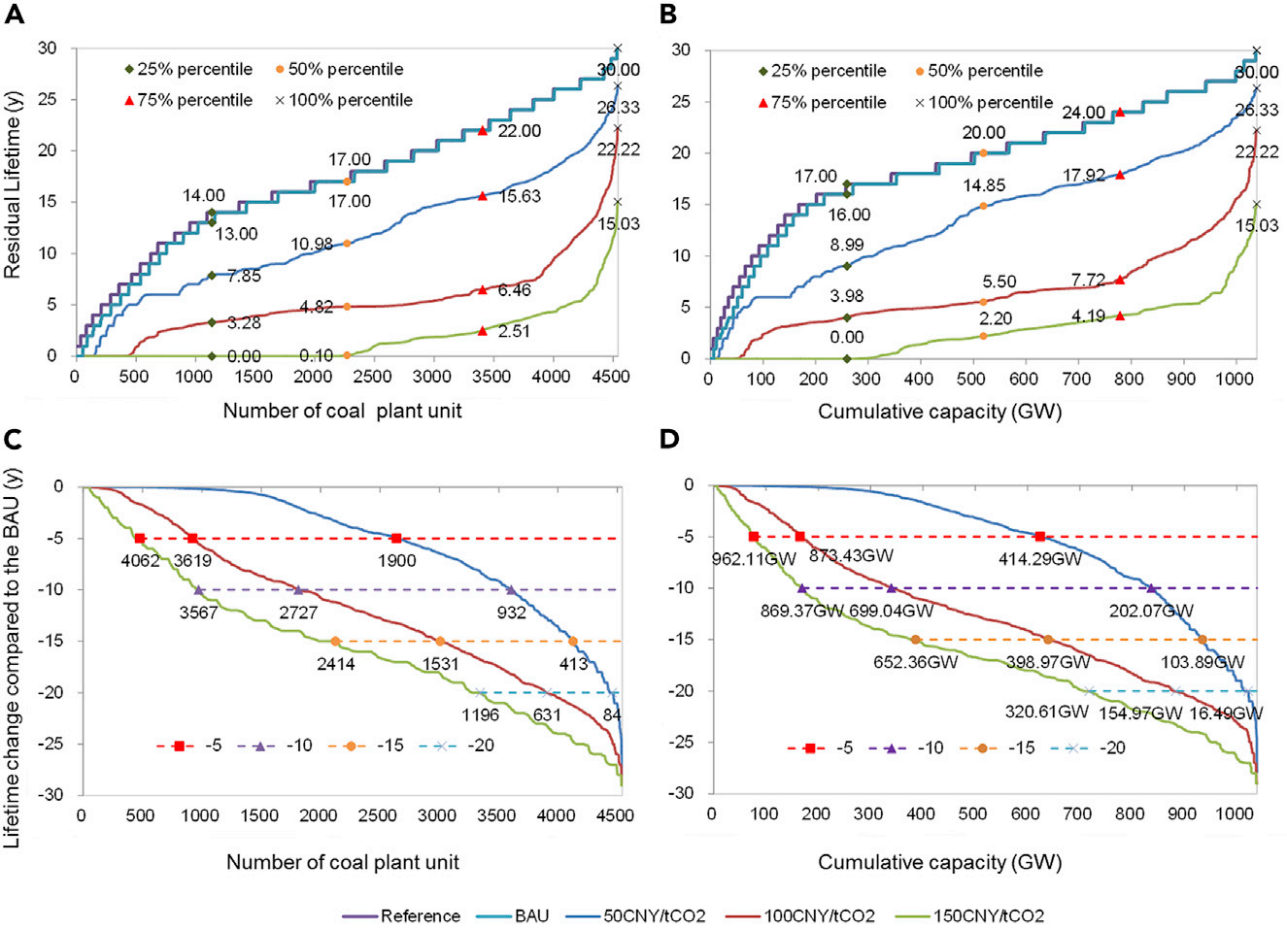
The residual lifetime of 4,540 power plant units and the corresponding lifetime change induced by carbon pricing Panels (A) and (B) show the residual lifetime distribution of the operating coal plant units, in which the label data demonstrate the percentile value of the residual lifetime; panels (C) and (D) show the distribution of the lifetime change relative to the BAU induced by carbon pricing, in which the label data demonstrate the number (C) and capacity (D) of the plant units whose residual lifetime is reduced by more than 5, 10, 15, and 20 years.

The probability distribution over time for each plant unit to become decommissioned (supplemental information, Figure S4) is obtained, based on which the residual lifetime of all plant units in different scenarios are calculated (supplemental information, Figure S5), and the overall distribution of the residual lifetime is shown in Figure 2. The technical lifetime of all the coal plant units is 30 years according to the operation plan designed at the initial stage, and in the Reference scenario without considering economic and policy conditions, all the plants will continue operating until the end of their technical lifetime. The results show that most of China's operating coal plant units are young and can operate for a long time in the future in the Reference scenario. Specifically, the mean and median values of the residual lifetimes of all the units are 17.28 and 17 years, respectively; 3,177 (70%) units, accounting for 870.50 GW (or 83.82% of total capacity), have a residual lifetime of more than 15 years, 1,731 units (38.22%) have a residual lifetime exceeding 20 years, accounting for 543.27 GW or 52.31% of the total capacity, and only 622 units (13.7%) or 77.93 GW (7.5% of the total capacity) will retire in less than 10 years. The results in the BAU market and policy conditions show the residual lifetime distribution without carbon pricing, which is almost the same as that in the Reference scenario, with the mean and median values of the residual lifetime being 16.99 and 17 years, respectively, except that only a small number (89 or 1.96%) of units, accounting for 17.47 GW (or 1.68% of the total capacity), have their residual lifetime reduced by more than 1 year.

With the average carbon price of 50 CNY/tCO_2_ observed in China's carbon emission trading pilots being introduced, the mean and median values of the residual lifetime of all the units are 11.56 and 10.98 years, which are reduced by 5.43 years and 6.02 years, respectively, corresponding to 18.10% and 20.06% of the plant lifetime in the Reference scenario. More specifically, 1,900 units (41.85%), accounting for 414.29 GW (39.89% of total capacity), will be closed down more than five years earlier than their normal end of lifetime in the BAU scenario; the lifetime of 932 units (20.53%), accounting for 202.07 GW (19.47% of total capacity), will be shortened by more than ten years; and those of 413 units (9.10%), or 103.89 GW (10.00%), will be shortened by more than fifteen years. Furthermore, as the carbon price increases to 100 CNY/tCO_2_, i.e., the high carbon price observed in Beijing and Shenzhen carbon trading pilots, the mean and median residual lifetimes of all the units are 5.26 and 4.82 years, which are reduced by 11.73 and 12.18 years, respectively, corresponding to 39.10% and 40.60% of the plant lifetime in the Reference scenario. In this situation, 3,619 units (79.71%), or 873.43 GW (84.10%), of the coal plants will be closed down more than five years earlier than the normal end of their lifetime in the BAU scenario; 2,727 units (60.07%), or 699.04 GW (67.34%), of the coal plants will be closed down more than ten years before their normal end of lifetime; and 1531 units (33.72%), or 398.97 GW (38.42%), of the coal plants will be closed down more than fifteen years before the normal end of their lifetime.

When the carbon price reaches 150 CNY/tCO_2_, the mean and median values of the residual lifetime of all the units are only 1.66 years and 0.10 years, respectively. Specifically, 2,182 units (48.06), or 267.04 GW (25.71%), of the coal plants will be closed down immediately after carbon pricing is implemented. Moreover, 75% of the coal plants in terms of unit number have a residual lifetime of less than 2.51 years, and in terms of capacity, 75% of the coal plants have a residual lifetime of less than 4.19 years. Thus, carbon pricing will have a significant effect on power plant operation in the future, and the residual lifetime of coal power plants will be shortened by the upcoming carbon pricing system.

### The evolution of the operating coal plant stock over time under carbon pricing

**Figure 3:**
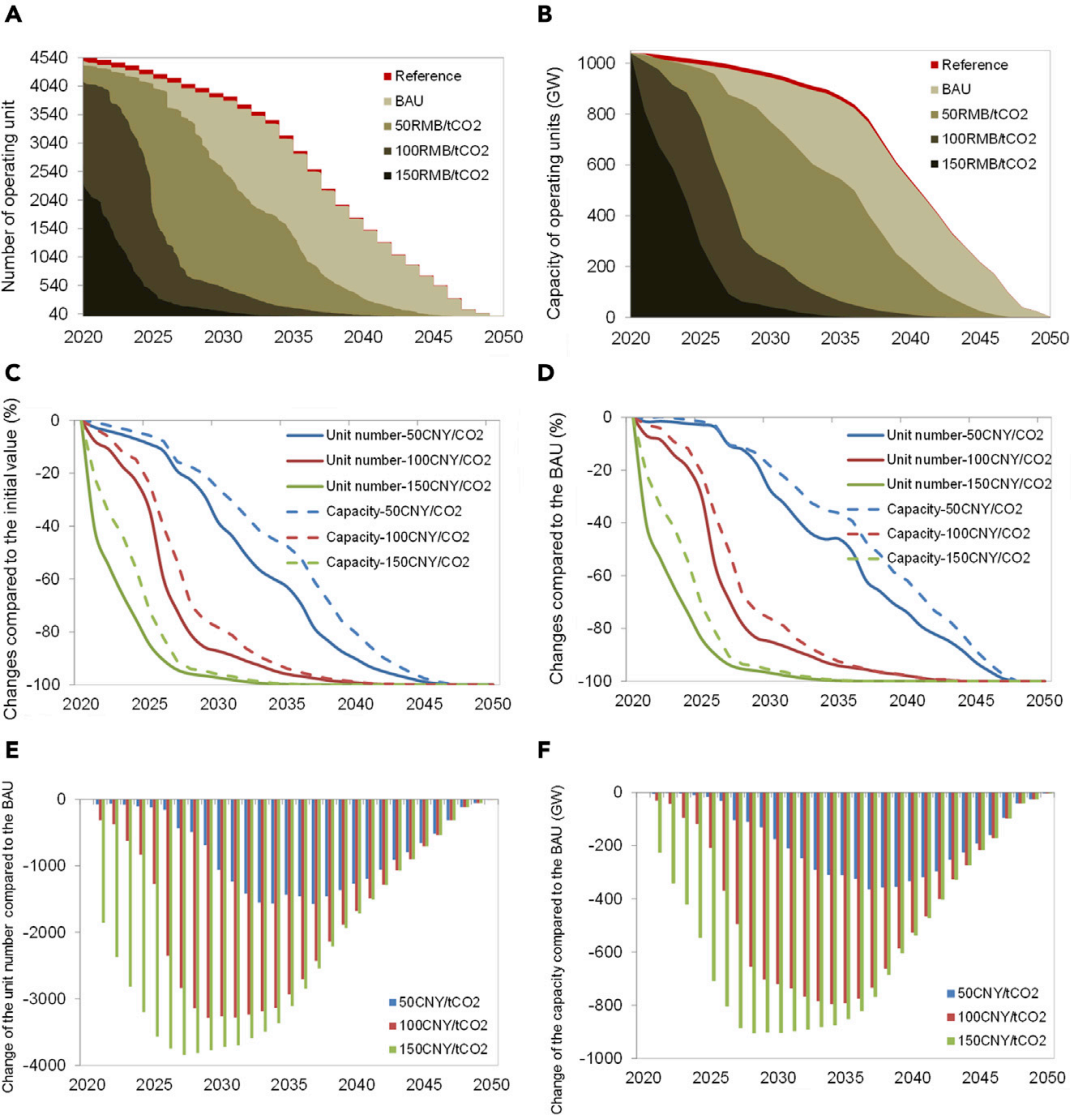
The evolution of the operating plant unit number and capacity over time in different carbon pricing scenarios Panels (A) and (B) show the evolution of the operating plant unit number and capacity over time; panel (C) shows the changes in the unit number and capacity relative to the initial values in 2020; panel (D) shows the changes in the unit number and capacity compared to the BAU results in terms of percentage value; panels (E) and (F) show the changes in the unit number and capacity in terms of absolute value.

As some of the coal plant units may be decommissioned before their normal end of lifetime due to carbon pricing, the plant unit number and capacity may decrease accordingly in the future, and the currently operating coal plant stock may be phased out earlier than that in the BAU scenario. As shown in Figure 3, the initial number of coal plant units was 4,540, and the total capacity was 1,038.57 GW at the beginning of 2020. In the Reference scenario, with some plant units being retired gradually as scheduled, they will decrease over time and will be halved by 2036 and 2039, respectively; finally they will reach zero by the end of 2050, when all the currently operating plants will have reached the end of their technical lifetime. In the BAU scenario without carbon pricing, the number of plant units and capacity are slightly fewer and less, respectively, than those in the Reference scenario before 2035, while after 2035, they are more or less the same in the Reference and BAU scenarios. Thus, it can be inferred that some old plants will be closed down before their normal end of technical lifetime, which is the main cause of the difference between the Reference and BAU scenarios at the early stage of the 2020-2050 period shown in Figures 3A and 3B.

With the carbon price of 50 CNY/tCO_2_ being introduced, the risk of coal plants being closed early will become higher, especially after 2025. Specifically, in 2030, the number of plant units and coal power capacity will be 2,550 and 720.61 GW, which are reduced by 43.83% and 30.62% compared to the initial values in 2020, respectively; in 2035, they will further decrease to 1,395 and 499.11 GW, corresponding to a decrease of 69.27% and 51.94%, respectively (Figure 3C). Furthermore, all operating coal plants will be phased out before 2048, i.e., 2 years earlier than in the BAU scenario. With the initial carbon price reaching 100 CNY/tCO_2_, the number of coal plant units will decrease rapidly and will be halved by 2023; 80% of the operating units will be closed down before 2027, and in 2044, all the plants will have been phased out, i.e., 6 years earlier than in the BAU scenario. Accordingly, the capacity will be halved before 2027; 80% of the capacity will be closed down before 2039, and all the existing plants will be phased out by 2044. Further, with the initial carbon price reaching 150 CNY/tCO_2_, in terms of the unit number, nearly 58% of the plant units will be closed down in 2021, and 98.87% of the plant units will be phased out before 2030. Correspondingly, in terms of capacity, 21.9% of the plants will be closed down in 2021, 68.3% of the plants will be closed down before 2030, and all the plants will be phased out before 2035, i.e., 15 years earlier than in the BAU scenario.

More interestingly, by comparing the change in the plant unit number and that in capacity over time, it is found that the unit number curve is steeper than the capacity curve (Figure 3C), and the number of coal plant units decreases faster than the coal power capacity with the implementation of the same carbon price. In other words, there seems to be a lag effect for the capacity change relative to the unit number change, which implies that the smaller units are prone to bear higher risk than the larger ones and to be closed down earlier. There are two possible reasons for this result. First, smaller plant units generally have lower efficiency and accordingly higher carbon intensity of power generation and then have to pay more for carbon emissions from generating each unit of power with the same carbon price being introduced; second, closing down a larger plant often leads to much higher sunk costs than closing down a smaller one, and the decision to decommission a larger plant may probably be delayed, even when faced with temporary loss in some periods.

We further demonstrate the effect of carbon pricing on the phase-out of coal power by comparing the results in the carbon pricing scenarios with those in the BAU scenario (Figures 3D–3F). With carbon pricing being implemented, the changes in the plant unit number and capacity in terms of absolute value first increase and then decrease over time (Figures 3E and 3F), while the relative change in terms of percentage value increases monotonically (Figure 3D). The effect of the carbon price of 50 CNY/tCO_2_ is not significant before 2025. Specifically, in 2025, the number of coal plant units decreases by 159, from 4,170 to 4,011, i.e., a 3.81% decrease, and the capacity decreases by 30.34 GW, from 987.17 GW to 956.83 GW, i.e., a 3.07% decrease; in 2030, the number of coal plant units decreases by 1,234, from 3,784 to 2,550, i.e., a 32.61% decrease, and the capacity decreases by 209.09 GW, from 929.70 GW to 720.61 GW, i.e., a 22.49% decrease; and in 2035 and 2040, the unit number decreases by 51.07% and 79.15%, and the capacity decreases by 39.32% and 67.49%, respectively. These results show that in the low-carbon-price scenario (i.e., 50 CNY/tCO_2_), although the risk of becoming stranded induced by carbon pricing is currently low and in the short term, the risk will rise in the mid- and long-term future. With increasing carbon prices, the effect of carbon pricing will arise in the short term; especially in the scenario of 150 CNY/tCO_2_, the unit number and capacity decrease rapidly immediately after carbon pricing is implemented.

### The impact of carbon emission permits allocation method on the phase-out of coal power

**Figure 4:**
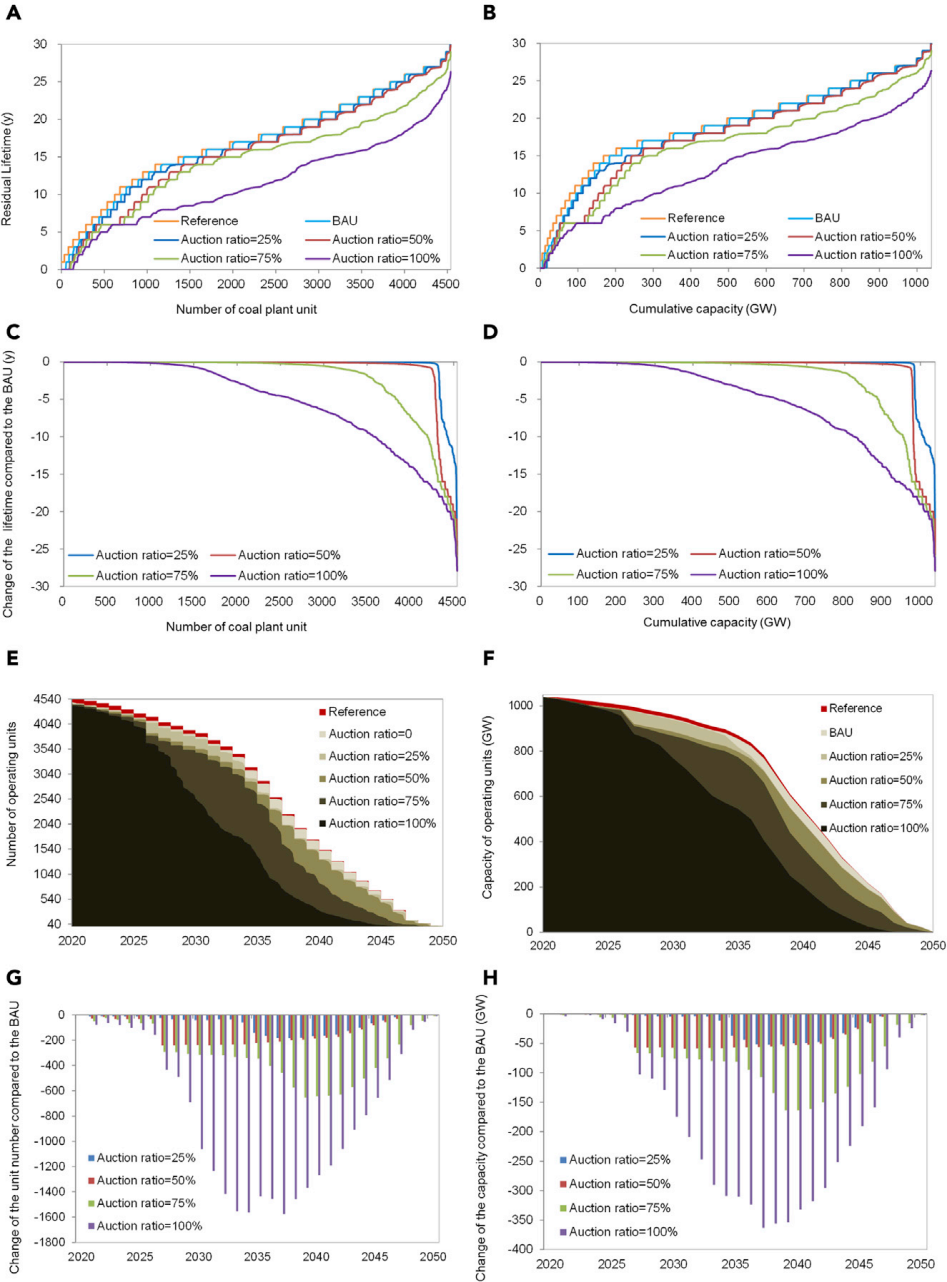
The evolution of the operating plant unit number and capacity over time with different carbon permit auction ratio Panels (A) and (B) show the overall distribution of the residual lifetime of all plant units; panels (C) and (D) show the distribution of lifetime change of all plant units; panels (E) and (F) show the evolution of the operating unit number and capacity over time; panels (G) and (H) show the changes in operating unit number and capacity relative to the BAU over time.

The carbon emission permits can be allocated to the coal plant manages by full auction (100% auction), partial auction or free allocation (0% auction) (Goulder et al., 2010), and the auction ratio determines how much coal plant managers have to pay to get the carbon emission permits, which will directly affect the cost of electricity production and then the coal plant operation decision. Figure 4A and 4B show the overall distribution of the residual lifetime of all plant units in different scenarios of the carbon permit auction ratio, with the current carbon price of 50 CNY/tCO_2_. As the auction ratio increases from 0 to 100%, the residual lifetime distribution curves move downward, and the residual lifetime gradually decreases. Specifically, with the auction ratio of carbon emission permits being 25%, 50%, 75%, and 100%, the average residual lifetimes are 16.48, 15.99, 14.70, and 11.56 years, which are reduced by 0.51, 1.00, 2.29, and 5.43 years, respectively; and the median residual lifetimes are 17.00 years, 16.93 years, 15.95 years, and 10.98 years, which are reduced by 0, 0.07, 1.05, and 6.02 years, respectively. Overall, the residual lifetime does not change much until the auction ratio reaches 50% and further increases.

To see more details about the lifetime change of all plant units, we also obtain the lifetime change distribution, as shown in Figures 4C and 4D. In the case of low auction ratio of 25%, only 4.80% of the units, corresponding to 5.70% of the total capacity, have their residual lifetime being reduced by more than 1 year. Similarly, even with 50% of the carbon permits being auctioned, only 6.37% of the units, corresponding to 7.36% of the total capacity, have their residual lifetime being reduced by more than 1 year. With the auction ratio further reaching 75%, 28.79% and 15.62% of the units' lifetime will be reduced by more than 1 year and 5 years, respectively. Thus the carbon pricing effect will not be significant until the auction ratio reaches 50%.

We also get the evolution of the operating plant unit number and capacity (Figures 4E and 4F), as well as their changes relative to the BAU results (Figures 4G and 4H) with different carbon permit auction ratios. As shown in Figure 4, the unit number and, especially, the capacity change little as the auction ratio increases before 2025. Specifically, with auction ratios of 25%, 50%, 75%, and 100%, the coal plant unit numbers are 4,077, 3,859, 3,809, and 3,668 in 2025, which are reduced by 0.61%, 5.92%, 7.14%, and 10.58% compared to the BAU results, and the capacities are 986.89, 985.76, 980.16, and 956.83 GW, which are reduced by 0.03%, 0.14%, 0.71%, and 3.07%, respectively. This indicates that in the short term, the auction ratio has little effect on the phase out of coal power. Even in the mid- and long-term, the unit number and capacity change little in the cases of low auction ratio (e.g., 25% and 50%), and the effect of carbon pricing is limited. Specifically, with the auction ratios being 25% and 50%, the unit numbers are reduced by 0.98% and 6.5% in 2030 compared to the BAU results, and the capacities are reduced by 0.48% and 6.36%, respectively; in 2040, the unit numbers are reduced by 12.24% and 13.56%, and the capacities are reduced by 10.57% and 11.18%, respectively.

### The carbon pricing effect on the CO_2_ emission

**Figure 5:**
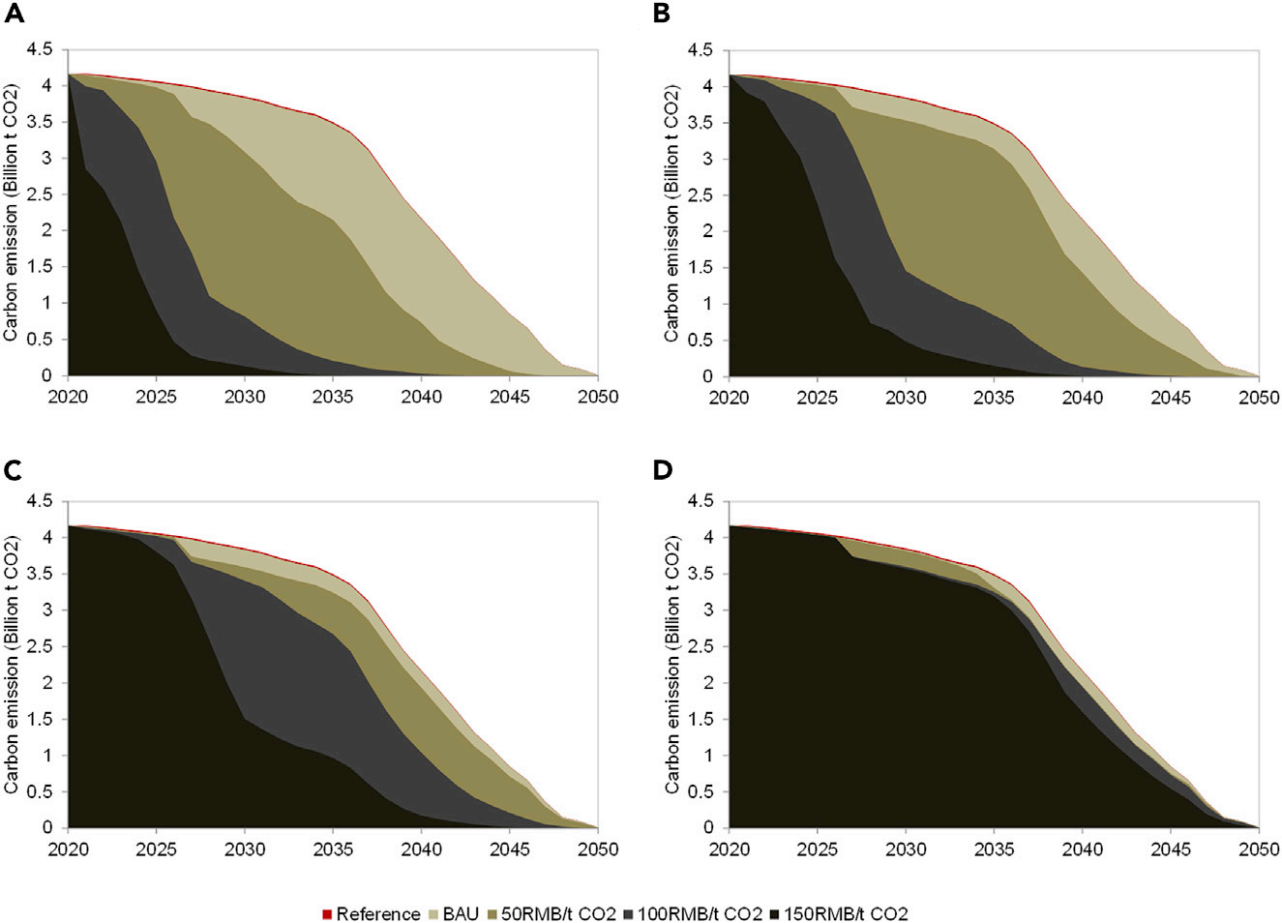
The expected carbon emission from the operating coal plants in the period of 2020-2050 Panels (A), (B), (C), and (D) show the carbon emission evolution with the carbon permit auction ratio being 100%, 75%, 50%, and 25%, respectively.

As mentioned above, with the carbon pricing being implemented, the coal power plant may be decommissioned earlier, and accordingly the carbon emission associated with the coal power generation will be reduced. As shown in Figure 5, the carbon emission will decrease gradually from 4.17 billion ton CO_2_ in 2020 to 3.48 billion ton CO_2_ in 2035, and further decrease rapidly to 0 in 2050. Accordingly the total carbon emissions from 2020 to 2050 are 84.73 and 84.38 billion ton CO_2_ in the Reference and BAU scenarios. As shown in Figure 5A, with the carbon pricing being implemented, the carbon emission will be reduced. Specially, if the carbon permits are allocated by full auction (Figure 5A), the total carbon emissions from 2020 to 2050 are 61.65 billion ton CO_2_, 31.33 billion ton CO_2_ and 15.54 billion ton CO_2_ with the carbon prices being 50 CNY/tCO_2_, 100 CNY/tCO_2_, and 150 CNY/tCO_2_, which are reduced by 26.93%, 62.87%, and 81.58% respectively compared with that in the BAU scenario. The auction ratio plays a key role in determining the effect of carbon pricing, and the carbon emission changes little with varying carbon price in the case of low auction ratio. Specially, with the auction ratio being 25% (Figure 5D), the total carbon emission are 82.09 billion ton CO_2_, 80.06 billion ton CO_2_, and 76.88 billion ton CO_2_ with the carbon prices of 50 CNY/tCO_2_, 100 CNY/tCO_2_, and 150 CNY/tCO_2_, which are only reduced by 2.71%, 5.12%, and 8.88% relative to that in the BAU scenario, respectively.

### The disparity of the carbon pricing effect on phase-out of coal power among China's 29 provinces, autonomous regions, and municipalities

**Figure 6:**
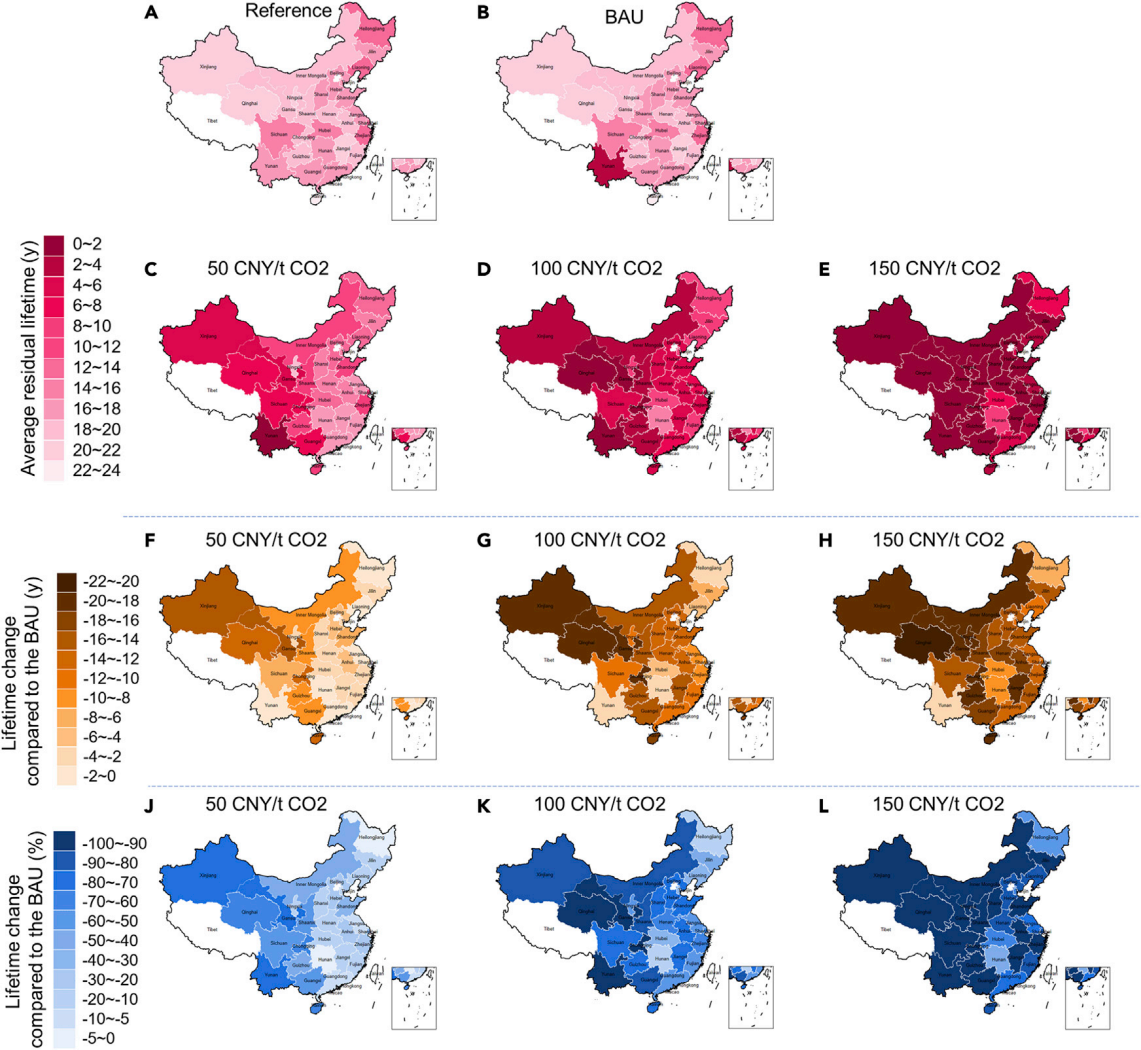
The residual lifetime of the operating plant units and lifetime change induced by carbon pricing in China's 29 provinces, autonomous regions, and municipalities Panels (A–E) show the average residual lifetime of the operating plant units in different scenarios of carbon pricing; panels (F–H) show the lifetime change induced by carbon pricing in terms of absolute value; panels (J–L) show the lifetime change induced by carbon pricing in terms of percentage value compared to the BAU without carbon pricing. Note: the data of the coal plant in Xinjiang are not available, and there is no operating coal plant in Beijing currently.

As a result of the disparities in the resource endowment, economic development stage, policy environment, etc., among different regions in China, the effects of carbon pricing on the coal plant phase-out in different regions likely differ, and quantifying this difference can help relevant stakeholders identify the regions that are vulnerable to carbon pricing risk. Thus, we categorize all 4,540 plant units into 29 groups according to which provinces they belong, and obtain the residual lifetime of each plant unit in these 29 provinces, autonomous regions, and municipalities (see more details in supplemental information, Figures S8 and S9), based on which we calculate the average residual lifetime of the operating plant units in each region under different carbon pricing scenarios. Accordingly, the changes in the residual lifetime induced by carbon pricing are obtained, which reflect the spatial disparity of the carbon pricing effect among China's 29 provinces, autonomous regions, and municipalities, as shown in Figure 6. In the BAU scenario without carbon pricing, the risk of coal plants' being decommissioned early is low in most of the 29 regions, and the residual lifetime in the BAU scenario is almost the same as that in the Reference scenario, except for Yunnan Province. Specifically, in the Reference scenario, the residual lifetime of all the plant units in Yunnan varies from 12 to 23 years, with an average of 17.86 years, while in the BAU scenario, the residual lifetime varies from 0 to 4.2 years, with an average of 2.61 years. This result indicates that even with no carbon pricing being introduced, plant managers in Yunnan have already faced much difficulty in continuing the plant operation, and it is optimal for them to decommission the plant immediately or in the short term. There are several reasons for this result. Yunnan has rich hydropower resources, and now, hydropower accounts for 71% of the total power capacity. In addition, the overall power demand has decreased during recent years, with China's economy entering into the phase of the “new normal”; in this situation, the competition between coal power and hydropower has become more intense. As hydropower has advantages over coal power in terms of marginal cost, average operating hours have been decreasing a great deal in recent years, totaling less than 2,000 hr in 2019. As a consequence, most of the coal power plants in Yunnan Province are bearing the brunt of these losses, and even with a low carbon price of 50 CNY/tCO_2_ being introduced, most of these plants are faced with the risk of becoming decommissioned immediately.

There are some provinces in which most coal power plants will continue operation in the BAU scenario but may be forced to close down much earlier than their normal end of lifetime, even with low carbon prices being introduced. Specifically, with the carbon price of 50 CNY/tCO_2_ being implemented, the average residual lifetime of coal power plants is reduced by 15.70, 14.32, 13.72, 13.57, and 12.04 years in Xinjiang, Gansu, Hainan, Qinghai, and Chongqing Provinces, respectively, which corresponds to 72.08%, 76.53%, 60.61%, 64.83%, and 60.58% decreases relative to those in the BAU scenario. This finding indicates that in these provinces, most of the plant operations are barely breaking even and are even operating at a loss with the current market and policy conditions, and a low carbon price shock may lead them to becoming closed down in the short term. Besides the provinces referred to above, Guizhou, Guangxi, Shaanxi, Inner Mongolia, Sichuan, and Shandong Provinces are also vulnerable to carbon pricing risk, and plants' residual lifetimes are very sensitive to carbon price variations. Specifically, the average residual lifetimes are reduced by 9.67, 9.51, 9.29, 8.10, 7.64, and 6.02 years, respectively, corresponding to 48.83%, 54.83%, 50.12%, 43.95%, 50.78%, and 35.77% decreases relative to those in the BAU scenario, and the risk of becoming stranded implied by carbon pricing is also high in these regions. In the other provinces, the residual lifetime of the power plants is shortened by less than 5 years, and the stranding risk in these provinces seems to be relatively low compared to the provinces referred above. Overall, the western regions will be more vulnerable to the carbon pricing risk than the eastern regions.

With the carbon price of 100 CNY/tCO_2_ being implemented, plant units will bear a higher risk of becoming stranded in most of regions. Especially in five provinces, i.e., Qinghai, Chongqing, Xinjiang, Gansu, and Hainan Provinces, the average residual lifetime of all the plant units will be shortened by more than 15 years, i.e., 19.66, 19.13, 18.75, 18.58, and 17.42 years, respectively; in 14 provinces, the residual lifetime will be shortened by between 10 and 15 years; in 6 provinces, the residual lifetime will be shortened by between 5 and 10 years; and only in 4 provinces, the residual lifetime will be shortened by less than 5 years. Furthermore, with the carbon price reaching 150 CNY/tCO_2_, the residual lifetime will be shortened by more than 15 years in 17 provinces, by between 10 and 15 years in 8 provinces, by between 5 and 10 years in 3 provinces, and by 2.6 years in only one province, i.e., Yunnan Province, corresponding to a 92.0% decrease relative to that in the BAU scenario.

## Discussion

Our results show that although China's operating coal plant units are young and have a long residual lifetime, many of their operations are close to the break-even state with the current policy and market conditions; the newly introduced carbon pricing may become “the straw that broke the camel's back”. Specifically, even with the implementation of the carbon price of 50 CNY/tCO_2_, i.e., the average carbon price observed in China's emission trading pilots, rising at 4% per year, the mean residual lifetimes of all 4,540 units are reduced by 5.43 years, and accordingly the carbon emission from 2020 to 2050 would be reduced by 22.73 billion ton CO_2_. Furthermore, with the carbon price increasing to 100 CNY/tCO_2_, i.e. the high carbon price observed in the Shenzhen and Beijing emission trading pilots, the mean residual lifetime is reduced by 11.73, and all the coal plant units will be phased out by 2044, i.e., 6 years earlier than without carbon pricing, with the carbon emission from 2020 to 2050 being reduced by 53.05 billion ton CO_2_. The results above have key implications for the relevant stakeholders of the coal power sector. In particular, potential investors should fully recognize this risk implied by carbon pricing and take it into account when making investment decisions. Not only does the premature retirement of the coal plant mean stranded assets and investment loss for investors, but it also consequently means bad debts and even systematic risk for the financial institutions if the scale of the stranded assets is large (Carattini and Sen, 2019). Financial institutions should be prudent when deciding whether to provide finance for carbon-intensive coal power investment. For the government, when they make future development plans (e.g., the 14^th^ Five Year Plan during the period of 2021–2025) for the coal power sector, they should also fully consider the potential risk of plant's becoming stranded, as closing down coal plants could also lead to mass unemployment (Burke et al., 2019) in the future. Moreover, the massive earlier retirement of coal power plants will generate tremendous stress on China's power sector, and much renewables will be required to replace such a large fraction of power generation in the next three decades. Thus how to keep the overall stability and reliability of electricity supply will be a great challenge, especially in view of that China's power demand growth will continue.

Our results also show that carbon permit allocation methods play a key role in determining the carbon pricing effect on the phase-out of coal plant stock. In the cases of low auction ratios, e.g., less than 50%, the effect of carbon pricing on stranding risk is limited, especially in the short term, and increasing the carbon permit auction ratio leads to a much higher stranding risk. In the practice of carbon emission trading, it generally starts with free allocation or a low ratio of carbon permit auctions to lower the resistance from the relevant industry, and the auction ratio may increase with the improvement of the system design in future, just as the EU ETS did. In this situation, the risk of coal plants' becoming stranded assets seems to be low in the initial stage and short term. However, the judgment should not be made just based only on the current policy and market conditions, and the possible adjustments of the carbon permit allocation policy in the future should be expected, so that the relevant stakeholders, especially the potential investors and financial institutions, can avoid making myopic decisions. From the perspective of the policy makers, if they want to rely on the carbon pricing to accelerate the coal power phase-out and energy transition, it's necessary to increase the carbon permit auction ratio.

The effect of carbon pricing on the phase-out of coal plants differs significantly among different regions, and the average residual lifetime will be shortened by between 0.34 and 15.71 years in China's 29 provinces, autonomous regions, and municipalities by the average carbon prices of 50 CNY/tCO_2_. Specifically, in Yunnan, Xinjiang, Gansu, Hainan, Qinghai, and Chongqing, the risk of becoming decommissioned early induced by carbon pricing is high. Xinjiang, Gansu, Qinghai, and Chongqing are all in western China, and the growth of local demand for electricity is limited compared to their large coal power capacities, which leads to the oversupply of electricity and low electricity pieces. In fact, a large portion of the power produced in Xinjiang, Gansu, and Qinghai is dedicated to be sent to the eastern regions of China, and the economic viability of coal plant operation in these regions depends much on the transmission network development, while the transmission capacity is still limited and should be improved in the future. In Gansu and Qinghai Provinces, renewable energy has developed rapidly in recent years, which has further worsened the situation of coal power development. Chongqing also has rich hydropower resources, which makes the oversupply of coal power worse. In Hainan Province, the coal power industry is facing great pressure, as Hainan Province is taking great measures to limit coal consumption to improve air quality. The relevant stakeholders of coal power should be prudent when evaluating future investment in coal power in the above regions.

### Limitations of study

In this work it is assumed that all the operating plant units are covered by the ETS, and the pace of coal power phase out may be faster than that in reality. In future work, how the coverage scope of the carbon pricing system would affect the pace of the coal power phase out can be further explored.

In addition, the carbon emission per year for each plant unit is determined by the coal power generation (or the operation hours) and its specific carbon emission (or the technology type of the unit), which are given exogenously. With the market-oriented reform of the power dispatch being implemented, the operation flexibility should be considered and then the carbon emission per year will be determined by the plant operator endogenously. In this situation, the effect of carbon emission benchmark on the results may become remarkable, which should be further explored.

With the coal power being phased out, more expensive renewable power may enter into the market gradually, and the electricity price may become higher. In this situation, the financial performance may be improved and the residual lifetime may become longer than that in our results. On the contrary, if the future renewable energy costs decrease rapidly, the future electricity price may become lower and the residual lifetime may be shorter. With the current partial equilibrium model, this effect cannot be explored, which can be done with general equilibrium model in future work.

As a key technology option to reduce carbon emissions, carbon capture and storage (CCS) may be a potential solution to hedging the stranding risk from carbon pricing. However, as a result of the disparity in plants' technical conditions, e.g., plant type, plant size, plant age, space available for CCS retrofits and CO_2_ storage, as well as the economic conditions, e.g., the CCS retrofit cost, capture cost, transportation cost, storage cost (Mo et al., 2015, 2018), etc., its viability remains to be further assessed. Especially considering that whether the operating coal plants can be retrofitted with CCS may be determined by the future carbon price, which will also be affected by the CCS development, this issue become more complex and should be further explored in future work.

## STAR★methods

### Key resources table

**Table:** 

REAGENT or RESOURCE	SOURCE	IDENTIFIER
**Deposited data**		

Note: The sources the complete data set can be found in Supplemental information (Table S3)		

**Software and algorithms**		

MATLAB	The MathWorks, Inc	R2021a

### Resource availability

#### Lead contact

Further information and requests for resources should be directed to and will be fulfilled by the lead contact, Jianlei Mo (mojianlei@casisd.cn).

#### Materials availability

The study did not generate new materials.

#### Data and code availability

The sources of the data sets supporting the current study have been presented. The other data and codes can be available on request from the lead contact.

### Method details

#### Modeling the decision to decommission a coal plant under carbon pricing

In this work, we develop a stochastic Monte-Carlo method to model plant managers' decisions regarding whether and when to decommission a plant under carbon pricing. Coal-fired power plants generally have a typical lifetime of 30 years or more, which exposes current investments and assets to future uncertainties in economic and regulatory conditions. The variation in input and output prices, and especially climate policy (e.g. carbon pricing), can have large impacts on the future cash flows and profitability of plant operations. Thus the electricity prices, fuel prices, and especially carbon prices, which reflect climate policy stringency in a carbon-constrained world, are the key drivers of the plant operation decision, and their dynamics are modeled using stochastic processes and numerically simulated using the Monte-Carlo method. Faced with the future uncertainties, plant managers can optimally decide whether and when to decommission the plant during the plant lifetime. As shown in Figure S1, assuming that the plant construction is finished and it starts operation in period $T_{0}$, the current period is $T_{1}$, and its technical lifetime will end in periodT. Thus the length of the whole plant's technical lifetime is ($T-T_{0})$, and the residual technical lifetime is $\left(T-T_{1}\right)$. In any periodt between $T_{1}$ and T, managers can choose the optimal timing of closing-down decision. By making a statistical analysis of the optimal timing on all the simulated paths, the expected residual lifetime of each plant unit can be calculated, and further by aggregating the residual lifetime of all the plant units together, when and how many of the operating coal plant units will be phased out can be obtained. The details about the model are as follow.

#### Market and policy dynamics

Managers of coal power plant face uncertain future costs and revenues, mainly because the future electricity market, fuel market, and especially carbon market are uncertain (Abadie and Chamorro, 2008). Although there is hardly a universal consensus on the stochastic process that best fits the behavior of coal prices, mean reversion has been frequently observed in a number of liberalized markets (Pindyck, 1999; Szolgayova et al., 2008; Benth et al., 2012; Nomikos and Andriosopoulos, 2012). China has made rapid progress in coal pricing reform, and the steam coal price has been mainly determined by the market since 2003, when government intervention in the steam coal market ended (Zhao et al., 2012; Liu et al., 2013; Cui and Wei, 2017). Thus, coal price dynamics are modeled using the mean-reverting process as follows,

(Equation 1) $$dP_{t}^{CO}=k_{CO}\left(L_{CO}-P_{t}^{CO}\right)dt+\sigma_{CO}P_{t}^{CO}dW_{t}^{CO}$$

where $P_{t}^{CO}$ denotes the coal price at time t. Parameter $L_{CO}$ stands for the long-term equilibrium coal price. $k_{CO}$ is the speed of reversion toward the equilibrium level.$\;\sigma_{CO}$ is the instantaneous volatility of the coal price. $dW_{t}^{CO}$ denotes the increment to a standard Wiener process.

Although China's electricity pricing is still under partial regulation, a new round of market-oriented electricity pricing reform was kicked off in 2015 via the issuance of the “No. 9 Document” (China State Council, 2015), and 18 provinces as pilots have been carrying out a market-oriented reform in China. It is expected that the electricity price will be increasingly determined by market force in the future, and the prices tend to revert toward levels of equilibrium in the mid- and long run, even with temporary deviation from the equilibrium. Thus, electricity price evolution is also modeled using the mean-reverting process, as was done in many other relevant studies (Abadie and Chamorro, 2008; Heydari and Siddiqui, 2010; Zhu and Fan, 2013).

(Equation 2) $$dP_{t}^{E}=k_{E}\left(L_{E}-P_{t}^{E}\right)dt+\sigma_{E}P_{t}^{E}dW_{t}^{E}$$

where $P_{t}^{E}$ denotes the electricity price at time t, while $L_{E}$ stands for the long-term equilibrium level to which the price tends in the long run. Parameter $k_{E}$ is the speed of reversion toward the equilibrium level.$\;\sigma_{E}$ is the instantaneous volatility of price, which determines the variance in $P_{t}^{E}$ at *t*. $dW_{t}^{E}$ denotes the increment to a standard Wiener process, which is normally distributed with a mean of zero and variance *dt*.

The current carbon prices in China's emission trading pilots are relatively low but are expected to increase gradually with the carbon mitigation opportunities being exploited and the carbon budget becoming more stringent in the mid- and long-term, reflecting China's commitment to taking on more ambitious carbon mitigation measures over time to achieve carbon neutral targets (Duan et al., 2018; Pahle et al., 2018). Moreover, the evolution of carbon prices is subject to many uncertain factors, reflecting uncertainties in both supply and demand factors that affect prices. We therefore model future carbon prices as a geometric Brownian motion (GBM) process, as was done in many other relevant studies (Abadie and Chamorro, 2008; Rohlfs and Madlener, 2013).

(Equation 3) $$dP_{t}^{CA}=\alpha_{CA}P_{t}^{CA}dt+\sigma_{CA}P_{t}^{CA}dW_{t}^{CA}$$

where $P_{t}^{CA}$ denotes the carbon price at time *t*. Parameter $\alpha_{CA}$ stands for the price drift rate. $\sigma_{CA}$ is the instantaneous volatility of the carbon price. $dW_{t}^{CA}$ denotes the increment to a standard Wiener process.

Furthermore, the risk-neutral form of the process is as follows:

(Equation 4) $$dP_{t}^{CA}=\left(\alpha_{CA}-\text{λ}\right)P_{t}^{CA}dt+\sigma_{CA}P_{t}^{CA}dW_{t}^{CA}$$

where λ is the risk premium, and $\left(\alpha_{CA}-\text{λ}\right)$ is the risk-adjusted price drift rate (Mo et al., 2015, 2018).

Finally, the increments of any two Wiener processes related to the price dynamics above may be correlated (Rohlfs and Madlener, 2013; Fuss et al., 2008), and we add the following equations to describe these correlations,

(Equation 5) $$\left\{ \begin{array}{l} dW_{t}^{E}dW_{t}^{CO}=\rho_{E-CO}dt\\ dW_{t}^{E}dW_{t}^{CA}=\rho_{E-CA}dt\\ dW_{t}^{CA}dW_{t}^{CO}=\rho_{CA-CO}dt \end{array}\right.$$

where the correlation coefficients are denoted by ρ, which reflects the extent to which both series move together beyond their trends, and a positive (negative) value for ρ implies that disturbances in one price are positively (negatively) reflected in the other price change.

Based on (Equation 1), (Equation 2), (Equation 3), (Equation 4), (Equation 5) above with specific parameter settings (see Tables S1 and S2), the future evolution of prices can be simulated using the Monte-Carlo method.

#### The decision on when to decommission a coal plant

As shown in in Figure S1, in any periodt between $T_{1}$ and T, if the future market and policy conditions are favorable enough to support continuing plant operation, then the plant manager may decide to continue operating the plant, leading to the continuation value $V_{t}^{C}$; otherwise, the plant manager may decide to decommission the power plant, leading to the residual value of the plant asset $V^{R}$. By comparing the two values above in each period of the residual technical lifetime, the plant manager can make optimal decisions regarding whether to continue plant operation or decommission the plant immediately: if the former is larger than the latter, then it is optimal to continue operating the plant; otherwise, the plant will be decommissioned immediately, leading to stranded assets. Accordingly, the plant manager can obtain the optimal project value from the optimal decision $V_{t}$:

(Equation 6) $$V_{t}=max\;\left(V^{R},V_{t}^{C}\right)$$

Specifically, the residual value of the asset $V^{R}$ is assumed to be a fixed proportion (β) of the original investment cost I (Zhao, et al., 2017),

(Equation 7) $$V^{R}=\beta I$$

The continuation value $V_{t}^{C}$ is composed of two parts: one is the immediate cash flow in period t $CF_{t}$, and the other is the discounted expected optimal value of the next period $V_{t+\Delta t}$, and it is calculated as their sum:

(Equation 8) $$V_{t}^{C}=CF_{t}+exp\left(-r\cdot \Delta t\right)\cdot E_{t}\left[V_{t+\Delta t}\right]$$

where Δt is the time length between any two successive periods, and r is the discount rate.

The net cash flow associated with the coal plant operation in period *t* ($CF_{t}$) is calculated as follows:

(Equation 9) $$CF_{t}=R_{t}^{E}-C_{t}^{F}-C_{t}^{CA}-C_{t}^{O\&M}-C_{t}^{T}$$

where $R_{t}^{E}$ is the revenue from electricity sales, $C_{t}^{F}$ is the fuel cost, $C_{t}^{CA}$ is the carbon emission cost, $C_{t}^{O\&M}$ is the operation and maintenance (O&M) cost, and $C_{t}^{T}$ is the tax cost. Specially, the carbon emission cost can be further calculated as follow,

(Equation 10) $$C_{t}^{CA}=\beta *P_{t}^{CA}*N_{t}^{CA}$$

where the $N_{t}^{CA}$ and the $P_{t}^{CA}$ are the carbon emission amount and the carbon price respectively, and β is carbon permit auction ratio. Further, the carbon emission of each plant unit in each period is calculated as follow,

(Equation 11) $$N_{t}^{CA}=CA*UH*CEG*\Delta t$$

where CA refers to the capacity of each plant unit, UH the annual utilization hours of a plant unit, and CEG the carbon emission factor of the electricity generation, which is determined by the plant type and technology, and Δt the time length of each period.

Then, the key to calculating $V_{t}^{C}$ is how to estimate the discounted expected optimal value of the next period, i.e., $E_{t}\left[V_{t+\Delta t}\right]$. To improve the accuracy of the estimation of $E_{t}\left[V_{t+\Delta t}\right]$, the least squares Monte-Carlo (LSM) method (Longstaff and Schwartz, 2001) is used. To be more specific, first we regressed the $V_{t+\Delta t}$ on a linear combination of a set of basic functions of stochastic variables, i.e., coal price $P_{t}^{CO}$, electricity price $P_{t}^{E}$ and carbon prices $P_{t}^{CA}$:

(Equation 12) $$V_{t+\Delta t}=a+bP_{t}^{E}+cP_{t}^{CO}+dP_{t}^{CA}+e{\left(P_{t}^{E}\right)}^{2}+f{\left(P_{t}^{CO}\right)}^{2}+g{\left(P_{t}^{CA}\right)}^{2}+hP_{t}^{E}P_{t}^{CO}+kP_{t}^{E}P_{t}^{CA}+lP_{t}^{CO}P_{t}^{CA}+\varepsilon_{t}$$

Then, we can estimate the parameters in the above equations and obtain the estimator of the parameters ($\hat{a},\hat{b},\hat{c},\hat{d},\hat{e},\hat{f},\hat{g},\hat{h},\hat{k},\hat{l}$) by using the least squares method. Relying on these estimated regression parameters and the simulated stochastic variables in period *t,* we can calculate the expected continuation value $E_{t}\left[V_{t+\Delta t}\right]$.

(Equation 13) $$E_{t}\left[V_{t+\Delta t}\right]=\hat{a}+\hat{b}P_{t}^{E}+\hat{c}P_{t}^{CO}+\hat{d}P_{t}^{CA}+\hat{e}{\left(P_{t}^{E}\right)}^{2}+\hat{f}{\left(P_{t}^{CO}\right)}^{2}+\hat{g}{\left(P_{t}^{CA}\right)}^{2}+\hat{h}P_{t}^{E}P_{t}^{CO}+\hat{k}P_{t}^{E}P_{t}^{CA}+\hat{l}P_{t}^{CO}P_{t}^{CA}$$

To check the robustness of the results, we also include the higher order of the stochastic variables. While this significantly increases processing time, the results change little.

The model is solved backwards from period T to the current period $T_{1}$, and the boundary conditions at periodT are as follows: if the plant is decommissioned immediately, then the manager can obtain the residual value of the asset $V^{R}$; otherwise, the manager can obtain the sum of cash flow from operating the plant in period T, $CF_{T}$, and the discounted residual value of $V^{R}$, and the optimal value of the coal plant asset in period T is as follows:

(Equation 14) $$V_{T}=max\;\left(V^{R},CF_{T}+exp\left(-r\cdot \Delta t\right)\cdot V^{R}\right)$$

By repeating the above decision in each period from the end of the technical lifetime T to current period $T_{1}$, the optimal timing ($t_{o}$) when to decommission the plant and the corresponding residual lifetime ($RLT_{t_{o}}$) can be calculated in each set of simulated price paths. Then, we compute the probability of decommissioning a coal plant unit at period $t_{o}$ ($Pro_{t_{o}}$) as the number of simulated paths on which a plant unit is decommissioned at the same period ($N_{t_{o}}^{S}$), divided by the total number of simulated paths N,

(Equation 15) $$Pro_{t_{o}}=N_{t_{o}}^{S}/N$$

Accordingly, the probability distribution (or the cumulative probability distribution) of becoming stranded over time can be obtained (as shown in Figure S4), and then, the expected residual lifetime of one plant unit ($RLT^{E}$) can be calculated as the probability-weighted average of all the possible residual lifetimes of this plant unit (as shown in Figure S5).

(Equation 16) $$RLT^{E}=\sum_{t_{o}}Pro_{t_{o}}*RLT_{t_{o}}$$

#### Parameters of market and policy dynamics

The parameters of the coal price (Equation (1)) and electricity price (Equation (2)) are estimated using the historical prices in China's 29 provinces, autonomous regions, and municipalities. The carbon price in the upcoming nationwide carbon market is the key driver of the decision to decommission a coal plant, and the most relevant issues for the plant managers are how the carbon price evolves and how the carbon emission permits are allocated to the plants, which directly determine the additional costs associated with electricity production and affect the coal plant operation in the future. The details on how to determine the relevant parameters are as follow.

#### Coal price and electricity price dynamics

We can estimate the parameters of mean reverting process for the coal price and electricity price using time series of historical prices following Abade and Camoro (2008). Here we take the estimation of the coal price process as an example to show the procedure of estimation.

After discretization of the mean-reverting process of Equation (1), we can get its discrete form as follow,

(Equation 17) $$\frac{P_{t+1}^{CO}-P_{t}^{CO}}{P_{t}^{CO}}=\left(e^{-k_{CO}\Delta t}-1\right)+L_{CO}\left(1-e^{-k_{CO}\Delta t}\right)\frac{1}{P_{t}^{CO}}+\sigma_{CO}\sqrt{\Delta t}\varepsilon_{t}^{CO}$$

where $\varepsilon_{t}^{CO}∼N\left(0,1\right)$.

The equation above can be further expressed as

(Equation 18) $$Y_{t}=a+bX_{t}+\mu_{t}$$

Where

(Equation 19) $$\left\{ \begin{array}{l} Y_{t}=\frac{P_{t+1}^{CO}-P_{t}^{CO}}{P_{t}^{CO}}\\ X_{t}=\frac{1}{P_{t}^{CO}}\\ a=\left(e^{-k_{CO}\Delta t}-1\right)\\ b=L_{CO}\left(1-e^{-k_{CO}\Delta t}\right)\\ \mu_{t}=\sigma_{CO}\sqrt{\Delta t}\varepsilon_{t}^{CO} \end{array}\right.$$

Then we estimate the parameters above by OLS method using a time series of 69 monthly average coal prices from January 2014 to September 2019, and consequently $k_{CO}$, $L_{CO}$ and $\sigma_{CO}$ can be obtained. As the 4540 coal plant units in our sample belong to 29 provinces, autonomous regions, and municipalities, we estimate the market parameters for each region of China. Similarly, the parameters for the electricity price (Equation (2)) in 29 provinces, autonomous regions, and municipalities can also be obtained following the procedure above. These parameters are presented in Table S1.

#### Carbon price dynamics

The historical carbon prices in the seven pilots vary from near zero to approximately 130 CNY/tCO_2_. With the implementation of the unified nationwide carbon pricing, the carbon prices in these pilots are expected to converge to some common level. Thus, the initial carbon price in the upcoming nationwide carbon market is set to 50 CNY/tCO_2_, which is more or less the same as the average carbon price in China's emission trading pilots; additionally, a scenario analysis of the initial carbon price level ranging from 50 CNY/tCO_2_ to 150 CNY/tCO_2_ is conducted.

In addition, carbon prices are expected to grow with the carbon mitigation target becoming more stringent and the low-cost carbon mitigation opportunities being exploited in future (Rohlfs and Madlener, 2013; Abadie and Galarraga, 2011). According to the relevant studies (Duan et al., 2018; Morris et al., 2019; Weng et al., 2018), the risk-free drift rate is generally set to 4%, and a scenario analysis of the risk-free drift rate varying from 1% to 7% was conducted to see how the carbon price drift rate or the growth rate affects the future evolution of operating plant stock (see Figure S6).

The historical prices in the carbon emission trading pilots are volatile, and the trading volumes are limited mainly as a result of the imperfect system design in the initial stage. It is expected that volatility may be gradually reduced and that prices may become more stable with the improvement of the system according to the experience of EU ETS (Abadie and Galarraga, 2011). We estimate the carbon price volatilities using the average daily carbon price of the emission trading pilots, which are 48.0%, 36.16%, 34.44%, and 30.69% in 2016, 2017, 2018, and 2019, respectively. In our evaluation, carbon price volatility is assumed to be further reduced in the upcoming nationwide carbon market in view of its larger coverage scope and improvement of policy design, and it is set as 15%, which is in accordance with some relevant studies (Liang et al., 2009). Additionally, a scenario analysis of the carbon price volatility varying from 20% to 30% is conducted to see how the carbon price volatility affects the future evolution of operating plant stock (see Figure S7).

The correlation coefficients cannot be estimated from historical prices, as the electricity market was still regulated during recent years by the administration, and the carbon market was still in its initial pilot stage. In this situation, their correlation cannot be reflected in the historical time series, as electricity producers were not able to pass on spikes in the carbon price to end consumers. However, with the deepening market-oriented reform of electricity pricing and the improvement of carbon market design, the pass-through between any two market prices will be realized. Here, we set the correlation coefficients following the relevant studies focusing on the EU ETS (Rohlfs and Madlener, 2011, 2013). Based on the above analysis, the parameters of carbon price dynamics in the upcoming nationwide carbon market are summarized and presented in Table S2.

In the practice of a carbon emission trading system, carbon emission permits can be allocated to the power plant by free allocation, partial auction, or full auction. Although more than 95% of emission permits are allocated by free allocation in the pilot stage, it is expected that the ratio of the auctioned permits in the nationwide carbon market will increase gradually in the future, in view of the international experience from the EU ETS. As we are concerned about the extreme risk induced by carbon pricing, all carbon emission permits are assumed to be allocated by full action, and a scenario analysis of the auction ratio varying from 0% to 100% is also conducted (see the results in Figure 4).

#### Data of China's 4540 operating coal power units

We collect detailed technical and economic data of 4,540 power plant units located in China's 29 provinces, autonomous regions, and municipalities and that account for 1,038.57 GW, i.e., nearly 100% of China's current operating coal power capacity. The built year ranges from 1990 to 2019, and all the coal plant units started operation during the period of 1991–2020. The technical lifetime of all the coal plant units are assumed to be 30 years according to the operation plan designed at the initial stage. Thus their residual lifetime vary from 0 to 30 years in the Reference scenario without considering economic and policy conditions, and accordingly, they will operate until between 2020 and 2050. More details on the distributions of existing coal-fired power plants in China regarding their location, built year, capacity, and technology can be found in the supplemental information (Figure S3).

For each plant unit, the costs and revenues associated with the coal plant operation are presented in Figure S2. The investment costs include the initial investment cost and the investment cost in ultralow emissions transformation. The fuel costs are determined by the plant unit's coal consumption of power generation and the coal price in specific regions. The carbon emission costs are determined by the plant unit's carbon emission factor, electricity generation, carbon price and the carbon permit auction ratio in the upcoming nationwide carbon market. The operational and maintenance (O&M) costs include fixed and variable costs. The fixed O&M costs further include repairing costs, issuance costs, maintenance material costs and labor costs; the variable O&M costs include plant unit modification costs, production material costs, water consumption cost determined by the plant unit's water consumption rate and the price of industrial water in specific regions, pollution control costs, pollutant emission costs for SO_2_, NOx, and smoke dust, and other variable costs. The tax costs include housing property tax, land-use tax, value added tax (VAT), urban maintenance and construction tax, education surcharge tax and VAT for water, fuel and materials. The revenues are mainly from electricity sales, and the relevant parameters include the plant unit's capacity, utilization hours, auxiliary power ratio and electricity prices in specific regions. All the included coal plant units started operation between 1990 and 2020, and their technical lifetime is assumed to be 30 years according to the plant operation plan designed in the initial stage; accordingly, the plants will continue operating until between 2020 and 2050.

We collect the relevant data referred above from multiple sources, and the details are shown in Table S3. Also we can use the current exchange rate (1 US dollars = 6.5 CNY) to calculate the costs and benefits in US dollars.

## Footnotes

Supplemental information can be found online at https://doi.org/10.1016/j.isci.2021.102655.
