# Supplementary material for: The role of national carbon pricing in phasing out China's coal power
Source: iScience. 2021 May 27;24(6):102655. doi: 10.1016/j.isci.2021.102655 (PMC8193610; doi:10.1016/j.isci.2021.102655)
Supplement: Document S1. Figures S1–S9 and Tables S1–S3 [file mmc1.pdf]

iScience, Volume *24*

Supplemental information

The role of national carbon pricing in phasing out China's coal power

Jianlei Mo, Weirong Zhang, Qiang Tu, Jiahai Yuan, Hongbo Duan, Ying Fan, Jiaofeng Pan, Jian Zhang, and Zhixu Meng

Supplemental Figures

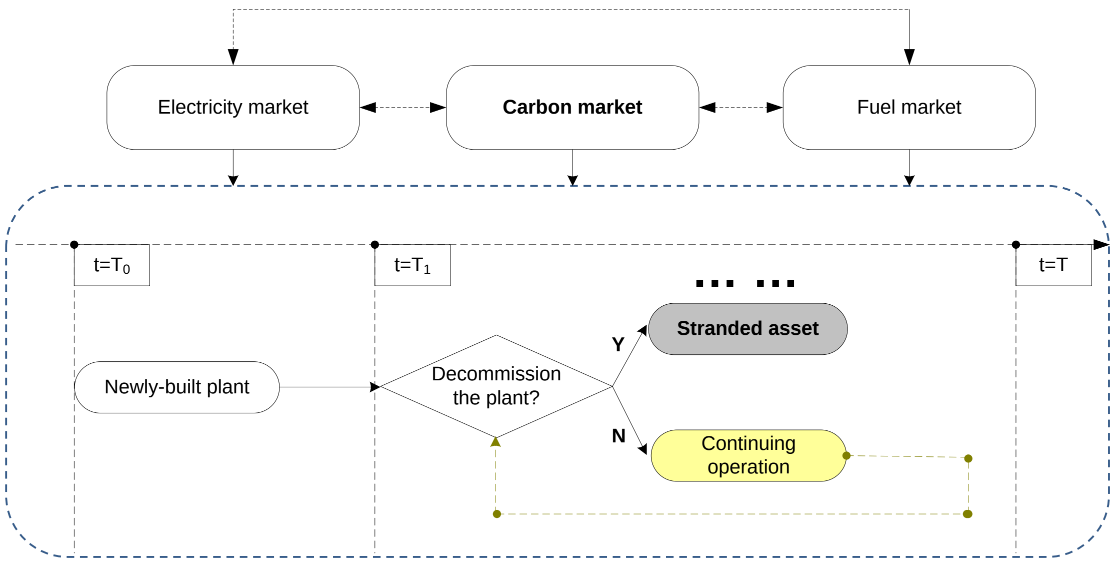

Figure S1. The plant manager's decision on whether and when to decommission a coal plant under carbon pricing, Related to STAR Methods.

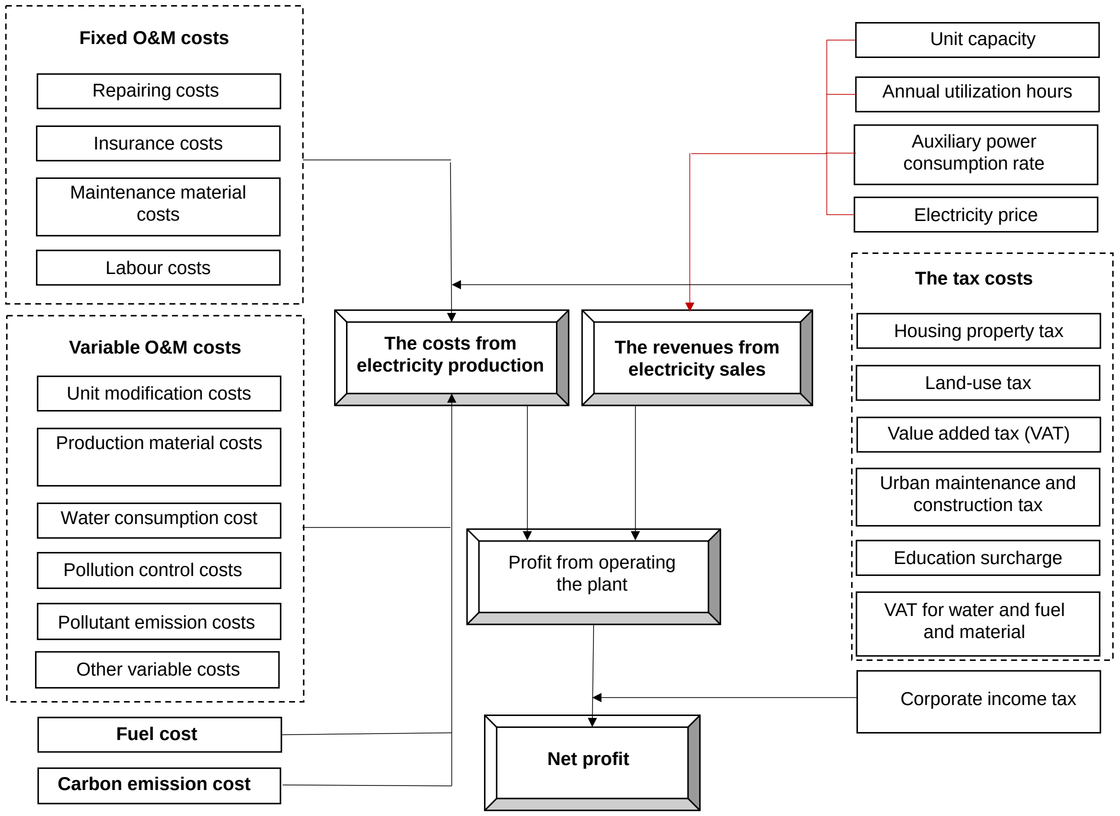

Figure S2. The costs and revenues associated with the coal plant operation, Related to STAR Methods.

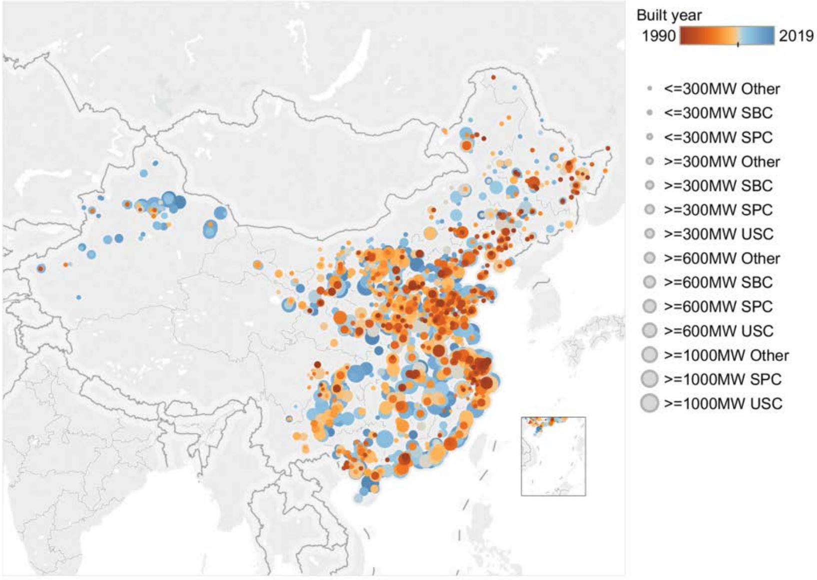

Figure S3. The distributions of operating coal plants in China regarding their location, built year, capacity, and technology type, Related to STAR Methods.

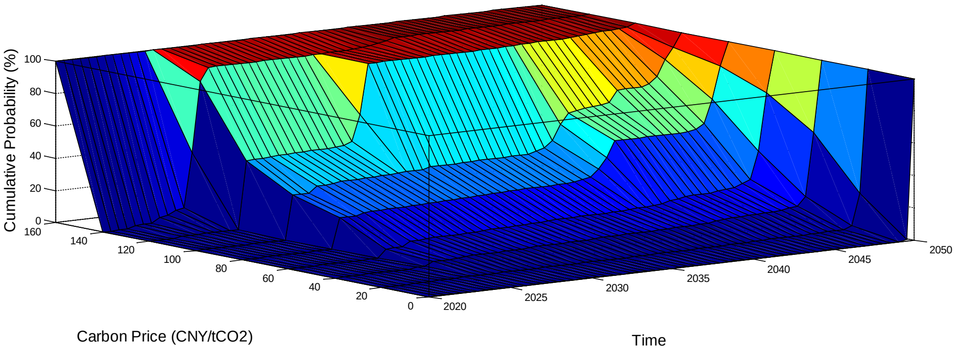

Figure S4. The probability distribution over time for a coal plant unit to become decommissioned in different carbon pricing scenarios, Related to STAR Methods and Figure 2. Here we take the results for one of the 4540 coal plant units as an example to show the risk distribution over time, and the results for the other plant units can also be obtained.

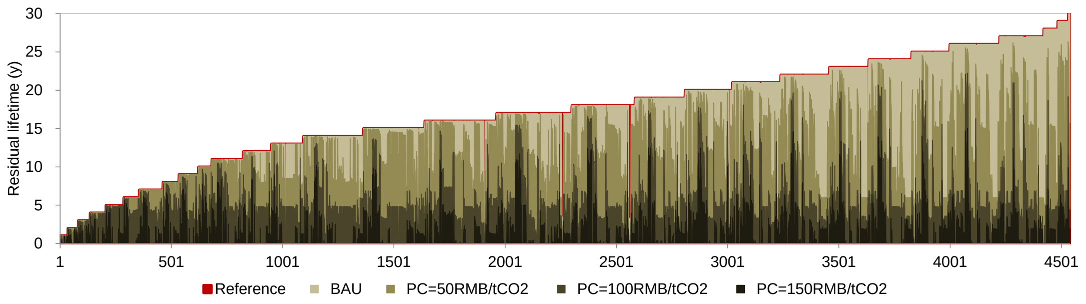

Figure S5. The residual lifetime of all the 4540 operating coal power units in different scenarios of initial carbon price, Related to STAR Methods and Figure 2.

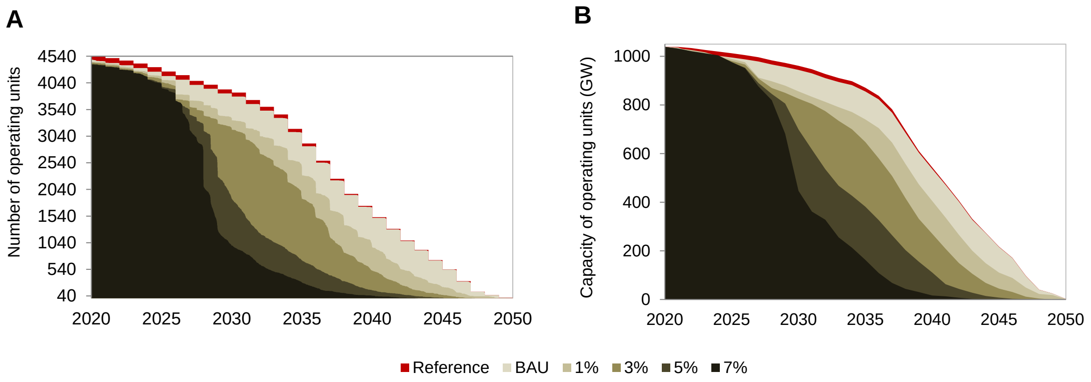

Figure S6. The impact of the carbon price growth rate on the future evolution of operating plant stock, Related to STAR Methods and Figure 3.

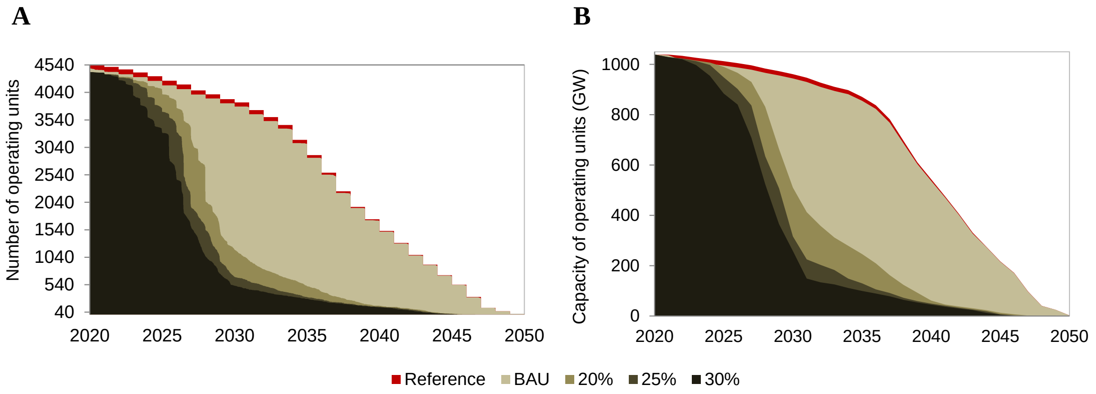

Figure S7. The impact of the carbon price volatility on the future evolution of operating plant stock, Related to STAR Methods and Figure 3.

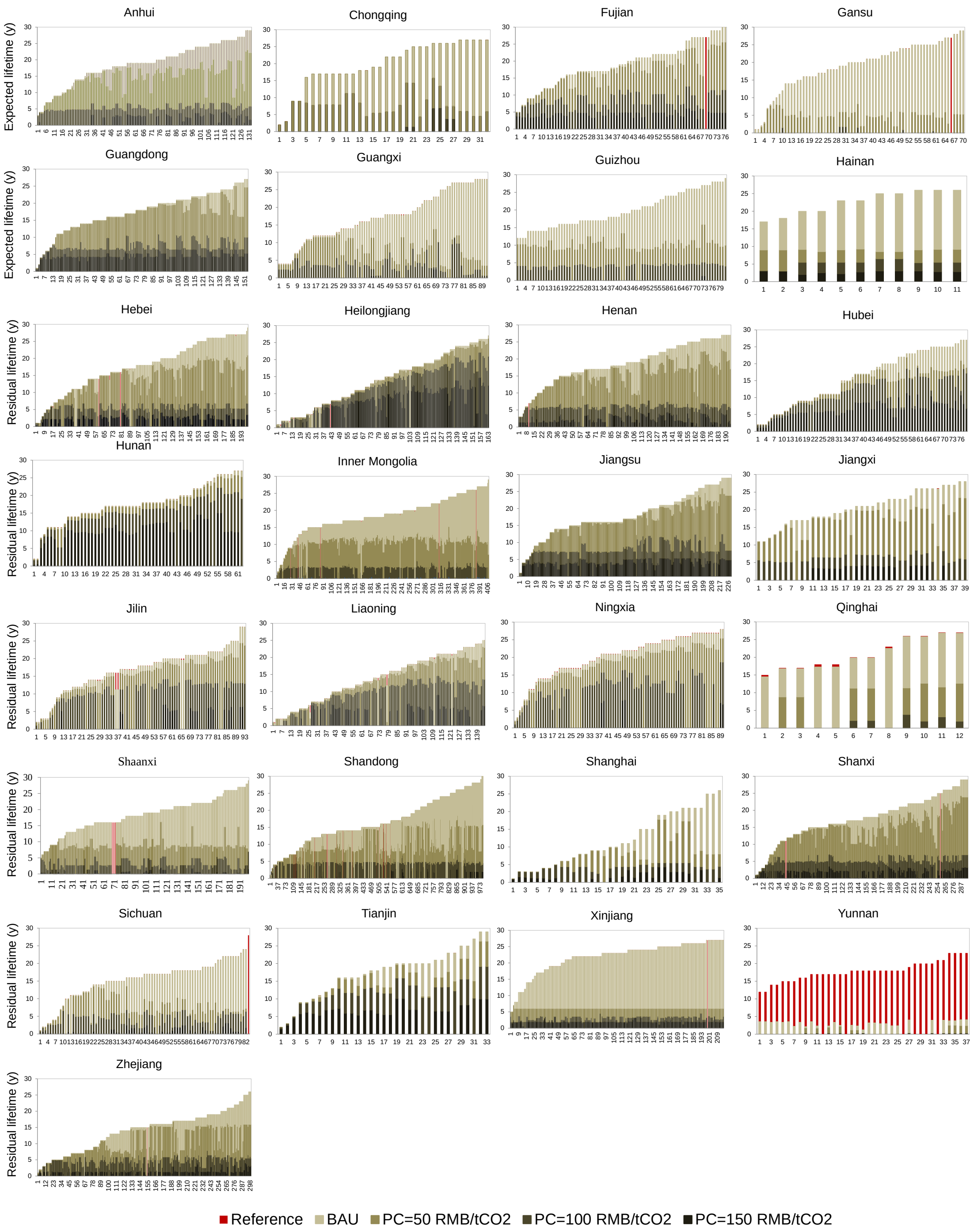

Figure S8. The residual lifetime of each coal power unit in China's 29 provinces in different carbon pricing scenarios, Related to Figure 6.

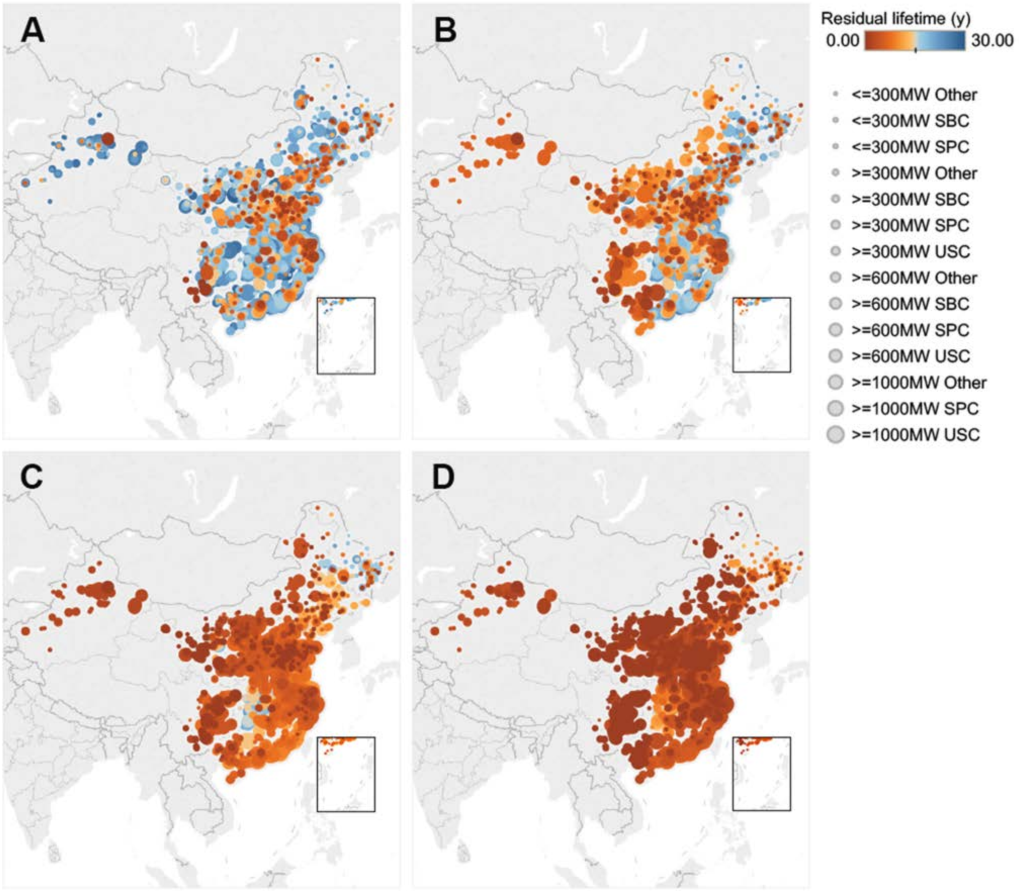

Figure S9. The cluster analysis of carbon pricing effect on the residual lifetime in terms of location, technology type, and capacity, Related to Figure 6. Panel (A) shows the results in the BAU scenario; Panel (B), (C) and (D) show the results in the scenarios of 50 CNY/tCO_2, 100 CNY/tCO_2 and 150 CNY/tCO_2.

Supplemental Tables

Table S1. The parameters of the electricity price and coal price in China's 29 provinces, Related to STAR Methods.

Region	P_0^E (CNY/kWh)	k_E	L_E (CNY/kWh)	σ_E	P_0^{CO} (CNY/t)	k_{CO}	L_{CO} (CNY/t)	σ_{CO}
**Anhui**	0.38	0.33	0.40	0.05	574.23	0.28	514.74	0.13
**Chongqing**	0.40	0.18	0.42	0.07	613.98	0.38	535.10	0.16
**Fujian**	0.39	0.49	0.41	0.06	532.02	0.27	508.47	0.13
**Gansu**	0.30	0.16	0.32	0.07	463.03	0.22	450.75	0.19
**Guangdong**	0.45	0.32	0.49	0.04	562.91	0.21	556.38	0.12
**Guangxi**	0.42	0.32	0.42	0.11	682.33	0.28	665.01	0.13
**Guizhou**	0.35	0.31	0.36	0.08	488.61	0.33	427.09	0.12
**Hainan**	0.43	0.30	0.45	0.06	505.27	0.65	498.82	0.13
**Hebei**	0.37	0.33	0.39	0.06	462.76	0.27	413.97	0.17
**Heilongjiang**	0.37	0.41	0.38	0.06	540.51	0.02	432.73	0.27
**Henan**	0.38	0.33	0.40	0.06	499.34	0.28	470.88	0.17
**Hubei**	0.42	0.44	0.41	0.11	583.65	0.28	511.49	0.15
**Hunan**	0.45	0.30	0.44	0.10	616.56	0.14	620.75	0.14
**Inner Mongolia**	0.29	0.36	0.25	0.05	283.04	0.15	299.11	0.11
**Jiangsu**	0.39	0.44	0.42	0.06	535.04	0.29	482.22	0.14
**Jiangxi**	0.41	0.29	0.43	0.07	649.13	0.27	585.21	0.12
**Jilin**	0.37	0.50	0.38	0.06	556.83	0.12	665.13	0.15
**Liaoning**	0.37	0.43	0.39	0.05	545.90	0.08	650.56	0.10
**Ningxia**	0.26	1.28	0.26	0.11	362.00	0.10	428.42	0.13

**Qinghai**	0.32	0.20	0.32	0.16	529.57	0.29	514.43	0.11
**Shaanxi**	0.35	0.22	0.37	0.07	393.92	0.39	357.36	0.16
**Shandong**	0.39	0.33	0.42	0.05	547.18	0.33	502.29	0.14
**Shanghai**	0.42	0.25	0.45	0.04	519.98	0.47	487.62	0.14
**Shanxi**	0.33	0.34	0.35	0.07	344.46	0.29	303.27	0.18
**Sichuan**	0.40	0.26	0.39	0.13	570.25	0.33	512.05	0.17
**Tianjin**	0.37	0.71	0.40	0.08	482.96	0.30	441.14	0.17
**Xinjiang**	0.26	0.14	0.26	0.02	258.74	0.34	202.00	0.13
**Yunnan**	0.34	0.21	0.34	0.11	457.68	1.55	458.71	0.20
**Zhejiang**	0.42	0.24	0.45	0.04	571.41	0.41	505.98	0.11

Table S2. Parameters of carbon price in the national carbon market and other key parameters involved in the evaluation, Related to STAR Methods.

Parameters	Value
P_0^{CA} (CNY/t CO_2)	50
$(\alpha_{CA} - \lambda)$ (%)	4
σ_{CA} (%)	15
ρ_{E-CA}	0.395
ρ_{E-CO}	0.6
ρ_{CA-CO}	-0.35
r (%)	5
Δt (y)	0.5
N	10000
β (%)	5

Table S3. The data sources of the technical and economic parameters of the coal plants, Related to STAR Methods.

Data	Source
Unit capacity, Time of starting operation, Plant type	Global Coal Plant Tracker (available at:: https://endcoal.org/global-coal-plant-tracker/)
Investment cost	Annual report of China power industry by China Electricity Council.
Repairing costs, Maintenance and material costs	China Huadian Corporation (available at: https://wenku.baidu.com/view/37d6087002768e9951e738db.html; https://wenku.baidu.com/view/7f663073f11dc281e53a580216fc700abb6852b0.html)
Insurance cost	Insurance Association of China (available at: http://www.iachina.cn/col/col4610/index.html).
Labour cost	Statistical Yearbook of China (2018), China Huadian Corporation (available at: https://www.docin.com/p-1680769165.html), State Grid (available at: https://wenku.baidu.com/view/b944b8ef81c758f5f61f6793.html) and China Power Investment Corporation (CPIC) (available at: https://www.docin.com/p-1961702978.html).
Specific coal consumption in power generation	China Electricity Council (available at: https://mp.weixin.qq.com/s/cp5hzbtcGcIIUShNkenVNQ).
Auxiliary power consumption rate	Annual Power industry statistics by China Electricity Council (https://www.cec.org.cn/index.html).
Fuel price	NDRC Price Monitoring Center (available at: http://jgjc.ndrc.gov.cn/).
Water price	China Water Net (available at: http://www.h2o-china.com/price/).
Unit modification cost	Polaris Power Net (available at: http://huanbao.bjx.com.cn/news/20160321/717860-7.shtml).
Pollution emission cost	Polaris Atmospheric Net (available at: http://huanbao.bjx.com.cn/news/20171103/859464.shtml).
Information of desulfurization and denitrification facilities of coal plant	the Ministry of Ecological Environment (available at: http://www.mee.gov.cn/gkml/hbb/bgg/201407/t20140711_278584.htm).
Tax costs	State Tax Administration (available at: http://www.chinatax.gov.cn/).
Annual utilization hours	China Electricity Council (available at:: https://www.cec.org.cn/menu/index.html?542).
Carbon emission factor for each type of coal plant	the Ministry of Ecological Environment (available at: http://www.mee.gov.cn/xxgk2018/xxgk/xxgk06/201909/t20190930_736483.html)
